# Recovering complete and draft population genomes from metagenome datasets

**DOI:** 10.1186/s40168-016-0154-5

**Published:** 2016-03-08

**Authors:** Naseer Sangwan, Fangfang Xia, Jack A. Gilbert

**Affiliations:** Biosciences Division (BIO), Argonne National Laboratory, 9700 South Cass Avenue, Argonne, IL 60439 USA; Computing, Environment and Life Sciences, Argonne National Laboratory, 9700 South Cass Avenue, Argonne, IL 60439 USA; Department of Ecology and Evolution, University of Chicago, 1101 E 57th Street, Chicago, IL 60637 USA; Department of Surgery, University of Chicago, 5841 South Maryland Avenue, MC 5029, Chicago, IL 60637 USA; Marine Biological Laboratory, 7 MBL Street, Woods Hole, MA 02543 USA

**Keywords:** Metagenomics, Genotype, Assembly, Binning, Curation

## Abstract

Assembly of metagenomic sequence data into microbial genomes is of fundamental value to improving our understanding of microbial ecology and metabolism by elucidating the functional potential of hard-to-culture microorganisms. Here, we provide a synthesis of available methods to bin metagenomic contigs into species-level groups and highlight how genetic diversity, sequencing depth, and coverage influence binning success. Despite the computational cost on application to deeply sequenced complex metagenomes (e.g., soil), covarying patterns of contig coverage across multiple datasets significantly improves the binning process. We also discuss and compare current genome validation methods and reveal how these methods tackle the problem of chimeric genome bins i.e., sequences from multiple species. Finally, we explore how population genome assembly can be used to uncover biogeographic trends and to characterize the effect of in situ functional constraints on the genome-wide evolution.

## Background

**Fig. 1:**
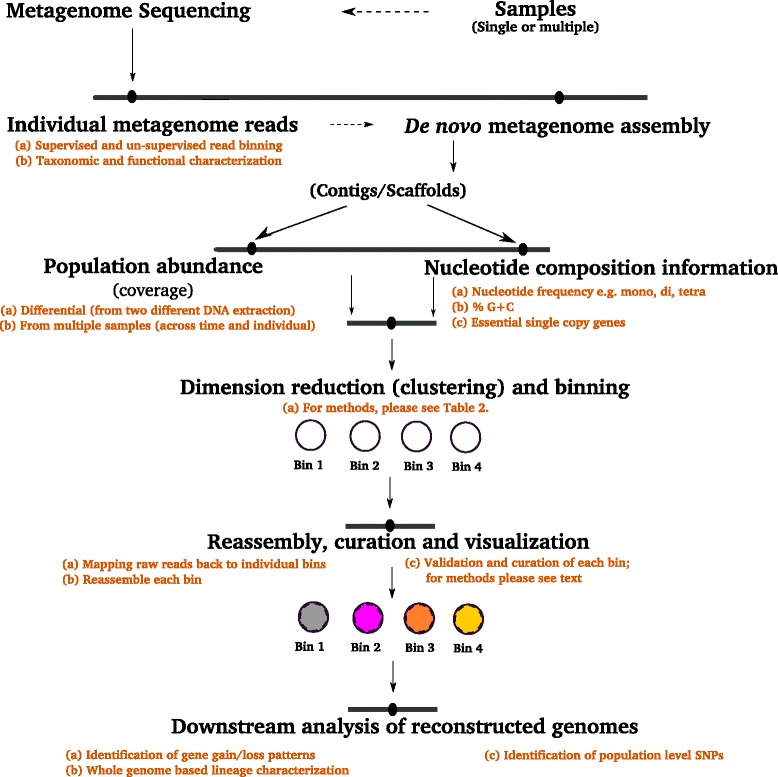
Workflow and overview for recovering population genomes from shotgun metagenomics data

Microbial ecology aims to understand the in situ microbial dynamics (taxonomic, functional, and evolutionary) of geochemically diverse environments, in part to elucidate how these environments select for particular microbial assemblages [1]. To characterize the microorganisms in these environments, we routinely employ metagenomic sequencing to predict the metabolic potential of the community of organisms without the need for prior cultivation. However, with the judicious use of metagenomic assembly tools, it is also possible to reconstruct the genomes of individual or closely related pools of microorganisms found in this community (Fig. 1). Through advances in computational infrastructure and software, we have seen a revolution in the use of metagenomic assembly to create a compendium genomes representing uncultured microbial lineages. Metagenomic studies of acid mine drainage channels [2, 3], the human gut [4], cow rumen [5], ocean environments [6], and bio-stimulated sediments [7] have demonstrated the utility of sequence assembly for the recovery of complete or draft genomes, including those of closely related organisms [8]. For example, metagenome (250 Mb) assembly was used to recover the genomes of two *Citrobacter* strains sharing ~99 % nucleotide identity with plausible genotypic variation in regulatory genes, flagella biosynthesis, and substrate metabolism [9]. These advances are revolutionizing the study of microbial ecology by enabling researchers to link the functional mechanisms that support specific metabolism with taxonomy and environmental context [10, 11].

Metagenomic recovery of complete or draft bacterial and archaeal genomes provides a route to analyze the “taxon-specific” potential of organisms within their community and ecosystem context. This is allowing insights into ecological adaptation, trophic interactions, and metabolic versatility of uncultured and eco-genetically adapted organisms (Fig. 1; [6, 12–16]). A genome can be defined as the total gene content of a single cell, whereas a population genome or genotype is defined as the total gene content of a group of closely related organisms. Genetic variability can be extensive in many bacterial species [17], which creates barriers to the recovery of strain-specific genotypes from complex microbial communities. This is because genome recovery (reassembly) methods are often based on clustering genetic sequences by nucleotide composition (oligonucleotide frequency-based correlation) and subsequent alignment-free visualization of metagenome contigs [6]. Therefore, within a population of extremely closely related strains, it is difficult to segregate the gene content of each genotype.

In this review, we discuss the existing theoretical frameworks and methodologies for reconstructing genomes from metagenomic data sets, how these methods are limited by the availability of computational resources, and provide a series of recommendations on best strategies for metagenome assembly (analysis of sequence coverage and assembly errors) and binning.

## Assembling contigs from short read metagenomic data

Assembling community genomics data, especially the co-assembly of multiple samples, is a complex task. This is in part due to computational memory constraints but mainly as a result of biological complexity, including genetic diversity and mobile genetic elements. Long stretches of near-identical metagenomic sequences are especially hard to assemble with the short reads from next generation platforms because such sequences could be from multiple sources: repetitive DNA of a single genome, homologous regions of closely related strains, or conserved regions of different species that coexist in the community. Failure to resolve these regions often result in rearrangement errors and chimeric assembly [18–20].

Recent developments in assembly algorithms [21–25] and related methods [26, 27] have led to significant improvements in the accuracy and efficiency of sequence assembly. These successes are measured by more contiguous pieces (greater N50 scores), increased numbers of predicted genes, fewer break points and rearrangements, and error limits close to the expected sequencing substitution error rate. Importantly, with memory use being such a bottleneck for analysis, many of these new programs have focused on reducing memory requirements, e.g., Minia [28], or parallel algorithms on distributed-memory machines e.g., HiPMer [29]. While metagenomic assembly is improving, metrics such as N50, designed for single-genome assembly, can be misleading; instead, combining N50 with other metrics such as rigorous statistical metrics e.g., ALE [30], fragment coverage distribution [31], total assembly size, and number of predicted non-redundant genes may provide an improved measure assembly success [27].

In 2012, Namiki et al. outlined the most important limitation of the single-genome assembly programs, namely, the inability of these algorithms to cluster sequence reads with diverse origins and heterogeneous coverage. Focusing on the “always increasing” property of the de Bruijn graph construction method, using the k-mer frequency patterns of the input dataset, the authors presented a strategy to decompose the de Bruijn graph of multiple species into subgraphs, each representing a cluster of reads from an individual species. However, since multiple species can have similar coverage patterns [32], these individual subgraphs can represent population genotype bins. A similar framework was implemented in various other metagenomic assemblers [24]. In 2012, Pell and colleagues [26] demonstrated the use of bloom filters as data structures for storing sparse sets as de Bruijn graphs (predominant assembly method), these filters lower the memory requirement by 40-fold. Recently, Scholz and colleagues presented a new method, metagenomic assembly by merging (MeGAMerge), to generate an improved metagenome assembly by merging the contigs generated from multiple assemblies [27]. Using an overlay consensus (OLC)-based assembler (for example, Minimus-2 [33]), contig bins assembled across different platforms (e.g., Velvet [34], SOAPdenovo2 [35], and Ray [36]), assembly parameters (e.g., k-mer length), and sequencing technologies (e.g., Illumina and Pyrosequencing) were merged into a composite assembly. Similarly, Deng et al. highlighted the sequential use of de Brujin graphs and OLC assemblers to increase the percentage recovery of targeted genomes [37]. Individual metagenome assemblies were generated from quality-trimmed metagenome reads (complete and partitioned datasets) using de Brujin graph-based assemblers such as SOAPdenovo2 [35] and ABySS [38]. Finally, multiple assembly outputs generated across complete and partitioned sequence datasets were merged using an OLC assembler CAP3 [39]. Optimized sequential use of different assembly platforms has demonstrated the potential to improve contig and scaffold lengths.

To determine the most effective strategy to use requires knowledge of how parameters such as genetic diversity, k-mer length, and sequence errors influence assembly success. To quantify these impacts, several recent studies have employed simulated shotgun sequence data [18, 40, 41]. Cahruvaka and Rangawala [41] suggest that the evenness of abundance of members of the community had a significant influence on accuracy, whereby the lower the evenness (greater dominance) the greater the accuracy; also, as expected, high intra-strain level diversity significantly reduced accuracy. They also demonstrated that clustering of contigs generated from assembly across different k-mer lengths created longer but less accurate contigs. Finally, while sequencing errors did not influence the annotation of gene function, they played a significant role in reducing the assembly accuracy [40]. However, to determine an effective strategy, one must also consider the metagenomic coverage, which is the fraction of total community diversity captured in the dataset.

Recently, Rodriguez and Konstantinidis highlighted methods for estimating metagenome coverage using real microbial metagenomic data [42]. Accurate coverage estimates are important in comparative studies as it informs the statistical tests required for interpretation of results [43] and is directly related to assembly quality [44]. Rarefaction analysis is the primary qualitative method used to estimate metagenome coverage, but it is suboptimal for metagenomic coverage analysis as it is reliant on deep sequence coverage of a metagenome, high-quality assemblies, and representative reference data sets, which limit its use for complex natural communities with low sequencing depth and for species with no reference genome [45, 46]. Nonpareil [42] addresses these problems by using singleton genes to calculate average metagenome coverage. Specifically, ungapped alignment between terminal regions of sequence reads is used to calculate the redundancy (portion of the total reads in the dataset that shows overlap with at least one other read) values for a subset of a complete dataset. Using a binomial distribution approach, individual read abundance (number of matches with other reads in the dataset) was processed to compute a saturation function of redundancy. Finally, the saturation function was summarized to calculate the average coverage. Nonpareil allows accurate estimation of the sequencing effort required to achieve a fixed average coverage, which can be used as a quality metric for the expected metagenome assembly.

## Binning: grouping assembled contigs into taxonomic bins

Metagenomic binning has two major components: (i) clustering and (ii) data representation. Clustering involves grouping contigs, scaffolds, or genes based on their genetic characteristics, including oligonucleotide frequency or coverage, using a combination of different approaches, such as hierarchical clustering and neural networks. These clusters are then grouped with various data representation approaches into individual taxonomic bins.

Based on the differences in sampling content (one sample or series of samples), clustering inputs (nucleotide composition-based or nucleotide composition-independent), and use of the abundance information, current methods of recovering genome bins from metagenome assemblies can be divided into three types (Table 1): (i) nucleotide composition (NC)-based, (ii) differential abundance (DA)-based, and (iii) nucleotide composition and abundance (NCA)-based. The major difference between the three methods is the starting point for the contig binning process. NC methods rely on oligonucleotide frequency variations. DA methods rely on the coverage of contigs across multiple samples where the organisms’ abundance changes. NCA-based approaches focus on creating a composite distance matrix from a combination of NC and DA analysis. It is worth noting that while earlier binning efforts were directed to raw reads, most pipelines today assemble them into longer contigs first. The reasons are as follows: (1) metagenome assembly used to be prohibitive and (2) NC and DA signals are both more pronounced and stable on longer sequences.

**Table 1:** Key methodological features of three main metagenome binning approaches

Method	Starting point	Clustering methods	Negatives	Positives	Computational Resources
Nucleotide composition (NC)	Oligonucleotide frequency matrix and %G+C-based screening.	HCL, correlation-based network graph and emergent self-organization maps (ESOM).	(i) More efficent for the genomes with skewed nucleotide composition patterns.	(i) Individual metagenome assemblies or samples where populations do not change over time can be used.	(i) R packages: qgraph (8), i graph, pv-clust [82]
					(ii) tetramerFreqs [83] (https://github.com/tetramerFreqs/Binning)
					(iii) Databionic ESOM tools [84]. (http://databionic-esom.sourceforge.net/)
			(ii) Less efficient in differentiating between closely related genotypes.		
					(iv) 2T-binning [85] (http://hmp.ucalgary.ca/HMP/metagenomes/data/SCADC/454/Binning/2TBinning/)
			(iii) Depends on the visualization and manual inspection of bins and therefore are not suitable for very large assemblies representing complex environments.		
Nucleotide composition and abundance (NCA)	A composite distance matrix from oligonucleotide frequency matrix and coverage.	K-medioids clustering, Gaussian mixture models, and expectation and maximization algorithm.	(ii), (iv) Require multiple samples for better performance, and therefore are associated with cost, time, and computational resources.	(i), (ii) Improved contig binning than NC method.	(i) MetaBAT [54]. (https://bitbucket.org/berkeleylab/metabat)
					(ii) CONCOCT [86] (https://github.com/BinPro/CONCOCT)
					(iii) MaxBin [87] (http://downloads.jbei.org/data/microbial_communities/MaxBin/MaxBin.html)
					(iv) GroopM [57]. (https://github.com/minillinim/GroopM)
					(v) Databionic ESOM tools [84] (http://databionic-esom.sourceforge.net/)
Differential abundance (DA)	Differential coverage patterns across multiple samples where population changed in abundance over time.	Profile based correlation cut-off.	(iv) Must have multiple samples with population changed in abundance over time, and therefore are associated with cost, computational time, and resources.	(ii), (iii) Strain level resolution can be achieved.	(i) Multi-metagenome [49] (https://github.com/MadsAlbertsen/multi-metagenome)
					(ii) MGS Canopy algorithm [51] (https://github.com/fplaza/mgs-canopy-algorithm).
					(iii) Databionic ESOM tools [84] (http://databionic-esom.sourceforge.net/)

The majority of the community genomics surveys published in recent years [3, 6, 12, 13, 16, 47, 48] have used NC, mostly oligonucleotide frequency and %G+C. Mackelprang et al. [13] used a hierarchical agglomerative clustering method to process the tetranucleotide frequency matrix and cluster metagenome contigs into genome bins, while Iverson et al. used a graph-based approach for assisting individual genome reassembly. In this latter study, a network graph was constructed where nodes (individual contigs and/or scaffolds) are connected by edges representing tetranucleotide Z-statistic correlation. Outliers were excluded from the graph using an empirically determined distance cutoff (Pearson’s correlation coefficient (PCC) >0.9). Connected nodes (scaffolds) of these graphs were later checked manually for coverage and %G+C profiles. Open-source software packages (Table 1) are available, including qgraph [8] and igraph (https://cran.r-project.org/web/packages/igraph/index.html), to perform such clustering and network-based graph construction and visualization. However, this pairwise analysis is computationally expensive for large datasets. NC techniques have mostly been applied to communities with genotypes that possess distinct nucleotide composition pattern, such as a low %G+C and consistent oligonucleotide frequency [6]. It is likely, though not proven, that this technique in isolation will struggle with communities that exhibit high oligonucleotide compositional variance.

In 2013, Sharon et al. demonstrated the DA approach on time series data to reconstruct six complete and two near complete bacterial genomes; these taxa had relative abundances as low as 0.05 % of the total community. The raw sequence data were assembled (de novo) using a de Bruijn graph approach, the contigs were binned according to the k-mer coverage, and the bins with greatest abundance were selected for the individual assembly. For each iteration, assembly parameters were optimized according to the selected coverage profile. Finally, the reads that mapped over the assembly were removed from the original set, and the remaining data was again binned according to the k-mer abundance to determine coverage. Size-selected (>3 kb) scaffolds were clustered into the bins using emergent self-organizing maps (ESOM) with a normalized time-series abundance profile.

A similar DA approach was used to recover the high-quality population genomes from environmental samples processed using two different DNA extraction methods, which resulted into the creation of two community gene pools with different population relative abundance profiles [49]. Size-selected scaffolds from the larger metagenome dataset were binned using coverage information, but then, tetranucleotide frequencies were employed for clustering and visualization (in a permutation on the NCA approach described below). Individual reads mapped over refined genome bins were extracted and reassembled independently. Paired-end information was further employed to identify multiple-copy genes, including rRNA operons. The authors also provided Perl scripts to facilitate the assembly visualization, including the reference-free assembly validation statistics [50].

Nielsen et al. used a DA method called Canopy to reconstruct microbial and phage genomes, and plasmids, using co-abundance patterns across multiple samples [51]. Initially, an iteratively optimized Markov clustering (MCL) algorithm and co-abundance-based correlation distance (1-correlation coefficient) matrix was used to cluster 2 % of the total community genetic repertoire with the strongest correlation to the human type 2 diabetes phenotype [52]. Again, due to the pairwise analysis, this method is computationally expensive, so Nielsen and colleagues used a novel approach to overcome these computational limitations. Using a global sequence identity cutoff of 95 %, a non-redundant community gene pool was created. Normalized co-abundance patterns were calculated for each gene using paired-end read mapping. Clustering was performed by randomly picking a “seed” gene from the community gene pool and cluster genes with similar co-abundance profiles using strict pairwise correlation cutoff. Each cluster then represents a “seed canopy,” and canopies with median abundance profiles within a distance of 0.97 PCC to each other and passing the rejection criterion explained in the original paper [51] were co-abundance groups (CAGs). CAGs with >700 genes were referred to as metagenome species (MGS), and reads mapped over MGSs were extracted from each sample and reassembled individually. This was referred to as MGS augmented assembly and resulted in the reconstruction of 741 genotypes including 238 microbial genomes meeting the high-quality genome standards of the Human Microbiome Project (HMP) [53].

In early 2014, a supervised binning NCA method called metagenome binning with abundance and tetranucleotide frequency (MetaBAT; [54]) was used to reconstruct 173 highly specific genome bins from a human microbiome metagenome collection. For each pair of contigs, MetaBAT calculates two probabilities of pairwise distances using tetranucleotide frequency and abundance patterns across samples. It then integrates all pairwise probabilities into a composite distance matrix. Using information from whole genome sequencing projects in IMG database, the authors suggest that sequencing bias can cause significant coverage variation among contigs assembled across one sequencing library. To overcome this coverage bias, a normal distribution-based approximation method was used to calculate the abundance matrix for each pair of contigs across one sample. Then, a geometric mean of all distances for all the samples was used to calculate the final abundance matrix. Finally, a modified k-medoid algorithm iteratively clusters the composite matrix into individual genome bins.

Due to the theoretical superiority of NCA methods, more tools (a binning algorithm without a cool acronym (ABAWACA) (https://github.com/CK7/abawaca), clustering contigs on coverage and composition (CONCOCT) [55], MaxBin [56], and GroopM [57]) have emerged in this category to provide automated genome binning. While all these tools bear family resemblance (e.g., some form of iterative clustering; the use of marker genes for bin delineation) to the MetaBAT algorithm described above as a representative, there are major modeling and algorithmic differences that are poorly understood. To date, our understanding of the impact of these differences comes from a small number of comparative evaluations by the method developers, and we have seen these tools give significantly different results on the same data set both in these experiments and in our own experience. Thus, we posit that the field of metagenome binning is in a similar place to where genome assembly or whole genome alignment was a few years ago before the occurrence of comprehensive benchmarks and competitive assessment studies such as Assemblathon [58], Alignathon [59], and GAGE [60]. An example of such comparative studies in binning (and metagenomic analysis in general) is the critical assessment of metagenome interpretation initiative (CAMI; http://www.cami-challenge.org) that is currently under way. Until we see outcomes of more external studies where unpublished, diverse, simulated, and real data sets are used for evaluating binning accuracy, it is unlikely that we will be able to conclusively recommend one tool over the others.

In the meantime, we have tabulated the key differences in the prominent NCA approaches (Table 2). It is important that we improve our understanding of how these design decisions affect binning accuracy. We highlight here the interesting algorithmic choices according to our intuition on how they manage to exploit more information than other approaches. (1) *Sequence composition model*: Most tools use tetranucleotide frequencies, but the dimension reduction in CONCOCT and GroopM allows them to be potentially more flexible with longer k-mers. MaxBin and MetaBAT do not use straight Euclidean distance but estimate probabilistic composition distributions from complete reference genomes. Of the two, MetaBAT’s model is more sophisticated as it accounts for different contig sizes. (2) *Differential abundance model*: Each tool computes coverage distance differently, and it is unclear which treatments are better. However, GroopM has shown in convincing visualization how uneven the coverage space is. Therefore, it may be advisable to transform coverage vectors for increased differentiating resolution in the crowded areas. (3) *Clusteringalgorithm*: Even though these approaches have different names for their clustering algorithms, most are variations of expectation-maximization algorithm, so they are more similar in nature than they seem. However, most tools have idiosyncratic ways of deriving the number of clusters without user intervention. ABAWACA is different from the rest in that it does not start with entire assembled contigs as the starting point. Rather, it breaks contigs into 5-kb fragments and self calibrates based on how these known groupings are recovered. (4) *Stopping criteria*: Most tools iterate until convergence or maximum rounds. GroopM has more custom substages than others. (5) *Post-processing and other notable heuristics*: Most tools check for genome completeness and chimeric assembly; some offer optional bin refinement. MetaBAT adjusts the weight of differential abundance progressively when more samples are available. CONCOCT combines compositional and coverage information into one vector that is used in Gaussian mixture models.

**Table 2:** Key methodological features of NCA-based metagenome binning tools

Binning software	Sequence composition model	Differential abundance model	Clustering algorithm	Stopping criteria	Post-processing and other notable heuristics
ABAWACA	Combined mono-, di-, and tri- nucleotide frequencies		Hierarchical clustering with iterative splitting; long scaffolds are broken into 5-kb fragments at the beginning; splitting based on a single metric that results in the best separation in each round	No separation can be made given quality score based on the extent to which the broken scaffolds are grouped correctly	Genome assessment based on marker genes and consensus taxonomic placement with reciprocal best BLAST hits; manual inspection using ggKBase; scaffold extension
Canopy^a^	Inter-assembly tetranucleotide frequency *z*-profiles created on 5-kb windows only in post-binning chimera detection	Abundance distance defined in terms of Pearson correlation and Spearman’s rank correlation coefficients	Canopy clustering (seed-and-recruit)	Stabilization of canopy profiles	Sample-specific augmented assemblies on two samples with most mapped reads and one with most gene containing de novo contigs
CONCOCT	K-mer frequencies (tetranucleotide by default); uniform Dirichlet distribution prior on the relative frequencies; dimension reduction using principal component analysis to keep 90 % of joined composition and coverage variance	Combined log-transformed profile of normalized coverage and composition vectors	Gaussian mixture models; regularized expectation-maximization; cluster number determined by automatic relevance determination	Parameter convergence and maximum iteration number	Empirical variational Bayesian approach; variational approximation used to perform integral in optimizing mixing coefficients
GroopM	Tetranucleotide frequencies; dimension reduction using principal component analysis to keep 80 % of compositional variance	Transformed coverage space to reduce unevenness of variability distribution	Iterative clustering in two custom steps: two-way clustering and Hough partitioning; bin refinement using self-organizing map	1:1 correspondence between bins and sub regions on the SOM surface	GC variance model for chimera detection
MaxBin	Tetranucleotide frequencies; Euclidean distance; empirically estimated Gaussian distributions of intra- and inter-genome distances	Poisson distribution	Expectation-maximization; cluster number estimated from single-copy genes; initial parameters inferred from the shortest marker gene	Parameter convergence and maximum iteration number	Recursive checking of all bins for median number of marker genes
MetaBAT	Tetranucleotide frequencies; Euclidean distance; empirical posterior probability derived from different contig sizes using logistic regression	Abundance distance defined as the non-shared area of two normal distributions	Modified K-medoid clustering without the need to set the number of clusters	Medoid convergence	Progressive weighting of the relative importance of DA vs TNF based on the number of samples; optional assembly, based on CheckM assessment, of mapped reads from a single most represented sample to reduce contamination

^a^We have also included the DA method Canopy because it uses sequence composition in post-binning refinement

The apparent orthogonal design considerations in the NCA tools lead us to think that the performance of these tools may depend heavily on the data and that one may achieve better results by combining multiple methods. Indeed, this is the lesson we learned in genome assembly: because there is no clear winner that suits all situations, ensemble approaches such as iMetAMOS [61], MeGAMerge [27], and GAM-NGS [62] were developed to try multiple assemblers on the same data or improve individual results by merging them. Given the a wide range of ecological diversity, sample numbers, and sequencing characteristics in metagenome data sets, we suspect that ensemble approaches will work best for genome binning as well.

There is also room for binning methods to improve in several directions. First, phylogeny information is still underexplored in automated NCA methods. Tetranucleotide frequency has been broadly adopted for its simplicity, but information theory-based studies show that relative k-mer abundance profiles may be better phylogeny signatures [63] and longer k-mers have higher information content [64]. Another source of valuable phylogeny signal comes from homology relationships between genes from assembled contigs and reference genomes. Traditional supervised approaches derive contig-level taxonomic placement from the consensus of individual predicted genes based on reciprocal BLAST hits. This method can be extrapolated to uncultured, unknown genomes without a close reference sequence [65, 66]. Despite the presence of horizontal gene transfer and uneven mutation rates along different protein lineages, there is a distinguishing power in the distribution of best hits across a range of diverse reference genomes. While this kind of information is leveraged by most research groups in post-binning inspection and refinement, incorporating it into automated optimization may greatly improve binning accuracy. Second, binning results are sensitive to parameters and most automated methods have limited preset parameters. This is one of the reasons achieving high-quality bins often requires manual tweaking aided by visualization. Automated parameter search needs to be part of ensemble binning methods, and computer vision-inspired algorithms such as Hough partitioning (used in GroopM) can also help further automate the curation process. Finally, most of the research in genome binning thus far has been concentrated on accuracy rather than computational efficiency. As a result, many binning tools are too slow or too memory intensive to handle large metagenomic data sets. Recent studies have changed the expectation for the number of bins from tens to hundreds [14, 65, 67]. As sequencing gets deeper, scalable tools such as MetaBAT and Canopy that are several orders of magnitude faster than other tools [54] will be appreciated.

## Curation and validation of reconstructed population genomes

Currently proposed methods for the validation of reassembled genomes rely on the same theoretical framework used for detecting misassembled regions and percentage completeness across individual genome assemblies. These include paired-end read mapping-based identification of misassembled regions (i.e., structural variations including deletions and insertions), alignment-based comparison with complete genomes of closely related reference organisms, and marker gene copy number variation analysis [13]. However, using paired-end mapping on sequencing libraries with the multimodal insert size distribution can increase the error rate, so that the number of false positive or negative events significantly increases. Meanwhile, alignment against reference genomes is fundamentally limited by the availability of already-sequenced genomes that are closely related to the organism of interest.

Two additional methods have been proposed to deal with the problem of chimeric genome bins (sequences from multiple species) observed in the metagenome assemblies [51]. First is identifying contigs with skewed coverage patterns; using peak detection methods, coverage subsets with more than one peak are selected and removed. Second is analyzing the nucleotide composition consistency in contigs with tetranucleotide usage patterns; a median tetranucleotide frequency *z*-profile can be calculated for each contig, and using an empirically determined cutoff for the Pearson’s correlation coefficient distance to this median profile, it is possible to cluster contigs into high-quality population genome bins.

Individual genotype fragmentation into two different bins can occur due to population level repeats, genome coverage, or sequencing %G+C bias [24]. To assess fragmentation, it essential to accurately quantify the genome bin completeness. The presence of single-copy genes, which mostly encode central metabolism processes (replication, translation, and transcription) or conserved core genes, are the primary target for assessing completeness. A set of 31 single-copy genes has been proposed for bacteria [68]. This was extended to the domain Archaea, and using reciprocal BLAST-based homology searches on 112,064 proteins from 50 representative archaeal genomes, 104 universally present, single-copy genes were identified [69]. Finally, a list of 101 hidden Markov models (HMM) from the Pfam [70] and TIGRFAM [71] databases has been produced that shows similarity to only one gene when compared against complete bacterial genomes (95 %; [72, 73]).

Recently, Parks et al. presented CheckM, a new method for estimating the completeness and contamination across population genomes [74]. Using marker genes that are specific to a genome-based lineage within a reference tree, CheckM provides better estimates of genome completeness and contamination compared to the universal single-copy marker genes. Similarly, Busco uses lineage-specific orthologs to estimate the completeness of the draft or complete genome [75]. However, using orthologous groups with single-copy orthologs in >90 % species (*n* = 40), Busco provides robust estimations across lineages with rare gene duplications and evolutionary loss of conserved genes, as is frequently the case of population genomes [65]. Overall, the probability of a universally single-copy ortholog being present in a single-copy genome is higher than a conserved marker gene, and thus, we advocate the use of CheckM.

## Using reconstructed population genomes to advance microbial ecology

Reconstructed population genomes can reveal how environmental factors shape niche-specific adaptations between individual taxa. In addition, they can also reveal the effect of in situ functional constraints on the evolution of microbial consortia. Comparative genomics of reconstructed genomes and their reference genomes employs analytical methods that are well understood and have been extensively reviewed [76]. However, another framework for analysis relies on variation in codon bias to determine the genome-wide influence of in situ functional constraints on individual taxa. Since percentage codon bias variation analysis is a phylogenetically independent method that directly reflects the strength of selection and the translation efficiency of expressed genes [77, 78], it circumvents the need for reference genomes and can reveal the influence of in situ functional constraints over natural selection patterns. It is important to note that for complete genome sequences, codon use patterns are influenced by nucleotide composition (mutational biases) and horizontal gene transfer. However, because each gene in a reassembled genome represents the population with an even nucleotide composition, one can assume that these clonal isolate-based limitations will not skew codon usage.

Recently, a near complete genome sequence (contigs = 200; 1.9 Mb) of a *Staphylococcus* genotype, *Staphylococcus* SK5, was reconstructed from a sample sequenced from the floor of a public restroom [48]. Whole genome-based average nucleotide identity (ANI) analysis revealed that SK5 shared strain level nucleotide identity (~99 %) with its ecotype from human-skin, *Staphylococcus lugdunensis* N920143 [79]. This suggested that this organism was dispersed from a human source and had potentially been selected for on the restroom floor. Furthermore, using pairwise codon bias variation analysis [50] across orthologous regions of both these ecotypes (N920143 and SK5), both genomes were observed to be under different environmental selection, suggesting that functional constraints dominated [48]. Similar evidence for considerable dispersal and environmental selection was observed in a sediment metagenome, from which a complete genome sequence (2.3 Mb) of a previously uncultured taxon, *Candidatus* Sulfuricurvum sp. RIFRC-1 [12], was recovered. Whole genome-based ANI analysis revealed that RIFRC-1 shares 75 % genome-wide identity with an ecotype from the oil fields of Japan, *Sulfuricurvum kujiense* [56]. Interestingly, comparing the codon bias variation across the orthologous segments of these ecotypes, it was clear that both populations were under similar and strong functional constraints. Using these approaches, it is possible to infer the mode of environmental selection for given taxa in specific ecosystems and hypothesize about potential effect of in situ functional constraints on the mutation pressure, natural selection, and genetic drift [77, 78, 80, 81].

## Conclusions

Recovery of novel genomes from metagenomic datasets provides components to better parameterize systems biology efforts, by increasing the availability of information on taxonomically resolved, novel metabolic potential. Also, using metagenome contigs binned at species level, phylogenetically independent analysis can be used to accurately estimate the strength of selection and translation efficiency of expressed genes assembled across ecotypes. The computational challenges that limit metagenomic-derived genome reconstructions are slowly being rolled back, and with the decreasing cost of high throughput sequencing, it will soon be possible to perform integrated analysis of inter- and/or intraspecies community dynamics with transcriptomic, proteomic, and metabolomic data from the same samples. Perhaps the most important implication of this integrative multi-omic analysis will be the potential to build predictive models that can further identify specific metabolic exchanges between species.

## Abbreviations

ABAWACA: a binning algorithm without a cool acronym
CONCOCT: clustering contigs on coverage and composition
DA: differential abundance
NC: nucleotide composition
NCA: nucleotide composition and abundance
